# Comparative Transcriptome Profiling of Two Tibetan Wild Barley Genotypes in Responses to Low Potassium

**DOI:** 10.1371/journal.pone.0100567

**Published:** 2014-06-20

**Authors:** Jianbin Zeng, Xiaoyan He, Dezhi Wu, Bo Zhu, Shengguan Cai, Umme Aktari Nadira, Zahra Jabeen, Guoping Zhang

**Affiliations:** Department of Agronomy, Key Laboratory of Crop Germplasm Resource of Zhejiang Province, Zhejiang University, Hangzhou, China; National Taiwan University, Taiwan

## Abstract

Potassium (K) deficiency is one of the major factors affecting crop growth and productivity. Development of low-K tolerant crops is an effective approach to solve the nutritional deficiency in agricultural production. Tibetan annual wild barley is rich in genetic diversity and can grow normally under poor soils, including low-K supply. However, the molecular mechanism about low K tolerance is still poorly understood. In this study, Illumina RNA-Sequencing was performed using two Tibetan wild barley genotypes differing in low K tolerance (XZ153, tolerant and XZ141, sensitive), to determine the genotypic difference in transcriptome profiling. We identified a total of 692 differentially expressed genes (DEGs) in two genotypes at 6 h and 48 h after low-K treatment, including transcription factors, transporters and kinases, oxidative stress and hormone signaling related genes. Meanwhile, 294 low-K tolerant associated DEGs were assigned to transporter and antioxidant activities, stimulus response, and other gene ontology (GO), which were mainly involved in starch and sucrose metabolism, lipid metabolism and ethylene biosynthesis. Finally, a hypothetical model of low-K tolerance mechanism in XZ153 was presented. It may be concluded that wild barley accession XZ153 has a higher capability of K absorption and use efficiency than XZ141 under low K stress. A rapid response to low K stress in XZ153 is attributed to its more K uptake and accumulation in plants, resulting in higher low K tolerance. The ethylene response pathway may account for the genotypic difference in low-K tolerance.

## Introduction

Mineral nutrition is crucial for plant growth and development. However, many plants are often subjected to nutrition stress due to insufficient nutrient supply in soils. Like nitrogen (N), potassium (K) is one of the most abundant elements in plants and performs vital functions in growth, stress adaptation and metabolism, as it is involved in stoma movement, enzyme activation, maintenance of cytosolic pH homeostasis, and stabilization of protein synthesis, etc [1]–[6]. Although K is quite abundant in the lithosphere and soils, being nearly 10 times higher than N and phosphorus (P) in terms of absolute content, most of them (90–98%) exists in the form of unavailability for plants [6], [7]. In other words, available potassium content in soils is commonly very low. In China, most soils show K deficiency for crops, and the case become more severe in recent decades, accompanied by a wide planting of hybrid rice, as it absorbs more K and is more sensitive to low K than inbred rice [8], [9].

On the other hand, plants have developed the strategies of coping with low-K stress. It has been well documented that there is a dramatic difference among plant species and genotypes within a species in the response to low-K stress [6], [7], indicating that K nutrition in plants is a genetically-controlled trait, and can be improved by genetic manipulation. Thus, it is imperative for us to reveal the mechanism or to explore the relevant genes of high K use efficiency. However, narrower genetic diversity in cultivated barley has become a bottleneck for genetic improvement [10]. The Tibetan Plateau is one of the centers of cultivated barley, and well known for its extreme environment [11]. The Tibetan annual wild barley (referred to wild barley thereafter) has been proved to be rich in genetic diversity and high tolerance to abiotic stresses, such as drought and salinity [12]–[14]. In the previous experiments, we found that wild barley grew well in the soils with poor fertility and less fertilizer application. Therefore it is possible that wild barley has the special mechanisms in tolerance to low-K stress.

Transcriptome analysis has been widely used in studies of functional genomics. There are two major approaches in the studies of transcriptomes, i.e. sequencing-based and hybridization-based. With the rapid advancement of High-throughput sequencing or so-called Next Generation Sequencing (NGS), RNA-Seq has recently become an attractive method. Compared with hybridization-based tool, such as microarray, RNA-Seq emerges as higher sensitivity, greater dynamic range of expression and base-pair resolution for transcription profiling [15]–[17]. Furthermore, it also shows clear advantages in revealing novel transcribed regions, single nucleotide polymorphisms (SNPs), the precise location of transcription boundaries and splice isoforms [18], [19]. This technique has been used in many plants to reveal gene annotation and expression under biotic and abiotic stresses [20]–[24].

Previous studies suggested that there is a considerable genetic variation in low-K tolerance among the wild barley accessions [25]. However, a comprehensive transcriptomic analysis of wild barley in response to low-K stress is still not done up to date. Based on the evaluation of low-K tolerance of 99 wild barley genotypes (accessions), we selected 2 wild barley accessions (XZ153, low-K-tolerant and XZ141, low-K-sensitive) as materials in transcriptome analysis using the Illumina RNA-Seq method. The objectives of this study are to determine (1) the possible difference in transcriptome profiles of two wild barley accessions in response to low-K stress; and (2) the signaling pathways and regulatory networks related to low-K tolerance.

## Materials and Methods

### Plant materials and low-K stress

A hydroponic experiment was conducted in a greenhouse with natural light at Zijingang Campus, Zhejiang University, China. Seeds of two wild barley accessions (XZ153, low-K-tolerant and XZ141, low-K-sensitive) were sterilized with 2% H_2_O_2_ for 30 min and rinsed with distilled water for three times, then soaked for 6 h at room temperature. The seeds were germinated on moistened filter papers in the germination boxes, placed into a plant growth chamber (22/18°C, day/night). Ten-days-old seedlings were transplanted into plastic pots (5L) for hydroponic incubation. The full-strength nutrient solution contains: 1 mM Ca(NO_3_)_2_.4H_2_O, 1 mM KCl, 1 mM MgSO_4_, 0.25 mM NH_4_H_2_PO_4_, 50 µM CaCl_2_, 20 µM Fe-citrate.nH_2_O, 12.5 µM H_3_BO_3_, 0.5 µM H_2_MoO_4_, 0.5 µM CuSO_4_.5H_2_O, 2 µM MnCl_2_.4H_2_O, 2 µM ZnSO_4_.7H_2_O. The pH was adjusted to 6.0±0.1 as required. Plants were supplied with half-strength of the hydroponic solution in the first week and then changed into full strength solution from the next week and renewed every five days. Three-leaf-stage seedlings were subjected to low-K treatment. The potassium concentration was adjusted to 0.01 mM (low-K treatment) and 1 mM as control, respectively.

### Biomass and potassium content determination

At 15 d after low-K treatment, the roots of all seedlings were thoroughly rinsed with tap water and dried with tissue papers. Then shoots and roots of seedlings were harvested and separated. All the plant samples were dried at 80°C for 72 h until their weight remained constant for biomass measurement. Dry shoots and roots were ground into powder, and approximately 0.1 g tissue sample was prepared for K content determination using Inductively Coupled Plasma-Optical Emission Spectroscopy (ICP-OES) (Optima 8000DV, PerkinElmer, USA).

### Gene expression assay

For time course pre-analysis of the expression of the gene *HvHAK1* under low-K stress, seeds of XZ153 were germinated and seedlings were cultivated as described above. All the endosperms were removed away from the seedlings to eliminate any additional supply of nutrition. The plants were incubated with 1/2 strength nutrient solution for 5 d and refreshed with full-strength for another 5 d. Then the two-leaf-stage seedlings were treated under low-K (0.01 mM) and normal K (1 mM) conditions. The roots of XZ153 were sampled with 3 biological replicates at 6 h, 12 h, 24 h, 48 h, 5 d and 7 d after treatment. The root samples were frozen in liquid nitrogen immediately and stored at −80°C for RNA extraction.

### RNA-Seq sampling and RNA isolation

For RNA-Seq sampling, seeds of XZ153 and XZ141 were germinated at the same condition and placed into a plant growth chamber. The two-leaf-stage seedlings were exposed to low-K stress (0.01 mM) for 0 h, 6 h and 48 h, respectively. Roots of 10 seedlings were collected and mixed together at each time point to reduce the differences between plant individuals. There were 6 samples [2 genotypes (XZ153, low-K-tolerant and XZ141, low-K-sensitive) ×3 time periods (0 h, 6 h, 48 h)] in total for further RNA-Seq research. RNA isolation was carried out according to the instructions of miRNeasy mini kit (QIAGEN, Germany). RNA abundances and purity was tested for meeting the requirements.

### Library construction, sequencing and data processing

mRNA enrichment was obtained from the total RNA by the magnetic beads with poly-T oligonucleotide. Then mRNA was randomly broken into fragments. Double-stranded cDNA was synthesized using reverse transcriptase combined with random primers and with adapters ligated at both ends. With those adapter sequences, DNA fragments were selectively amplified and enriched. Thus, the cDNA libraries were ready for sequencing. Qubit quantitation, insert size tested by Agilent 2100, and the Q-PCR were also conducted for accurate quantification before sequencing.

PCR products were loaded onto Illumina HiSeq2000 platform for 2×100 bp pair-ends sequencing. Then the RNA-Seq reads were generated via the Illumina data processing pipeline (version 1.8). To obtain the clean data, the raw reads were trimmed by removing empty reads, adaptor sequences and low quality bases at the 3 end. Then all the clean reads were considered for further analysis. The barley genome sequence and annotation data was downloaded, and TopHat (http://tophat.cbcb.umd.edu/) was adopted to align RNA-Seq reads to the barley reference genomes using the ultra high-throughput short read aligner, and then analyzes the mapping results to identify splice junctions between exons.

### Identification of the differentially expression genes (DEGs) and quantitative RT-PCR analysis

For gene expression analysis, the expression level of each gene was calculated by quantifying the number of reads. Gene expression counts were normalized by a variation of the FPKM (fragments per kilo-base of exon per million fragments mapped reads) method [26]. To identify differentially expression genes (DEGs) between the two different samples, the software Cufflinks was employed to output the T-statistic and the p-values for each gene [27]. We calculated the expression ratio of 6 h/0 h or 48 h/0 h as fold changes, respectively. Differentially expressed genes (DEGs) were required to have a 2-fold change and p≤0.01. In addition, an FPKM value≥2 in at least one of the samples was applied to genes for statistical analysis [28].

The RNA samples for RNA-Seq were also used for real-time PCR assays to confirm the reliability of the RNA-Seq result. 1 µg total RNA was treated with DNase I to eliminate the genomic DNA contamination, used as a template for reverse transcription (Takara, Japan). First strand cDNA was synthesized with oligo dT primer and Random 6 mers in a 20 µl reaction. Real time PCR was performed on a CFX96 system machine (Bio-Rad, USA). The PCR profiles were as follows: Pre-denaturation at 95°C for 30 s, 40 cycles of denaturation at 95°C for 5 s and annealing at 60°C for 30 s, followed by steps for Melt-Curve analysis (60°C–95°C, 0.5°C increment for 5 s per step). The relative expression of the chosen genes was calculated according to the comparative CT method [29]. In order to normalize all the data, the amplification of *HvGAPDH* sequence was used as endogenous reference. The gene specific primers were designed using primer-blast (http://www.ncbi.nlm.nih.gov/tools/primer-blast/). All the primers were listed in Table S6.

### Gene annotation, GO enrichment and KEGG analysis

The Blast2GO program was used to obtain GO annotation for the DEGs, as well as for KEGG analysis (http://www.blast2go.com/b2ghome) [30]. BLASTx was performed to align against NCBI non-redundant (nr) protein database for homology search. Following the mapping step, the gene ontology (GO) annotation, InterProScan annotation and enzyme code annotation steps were conducted in details with default parameters. The GOs distribution associated with DEGs were then obtained from three levels: molecular functions, biological processes and cellular components. The KEGG maps containing the EC numbers and enzymatic functions in the context of the metabolic pathways, in which they participate as well as annotation results can be available in a variety of formats [31].

### Statistical analysis

The significance of difference between the two barley genotypes in physiological traits and gene expression was examined using data processing system (DPS) statistical software package, followed by the Duncan's Multiple Range Test (DMRT) and the difference at P<0.05 and 0.01 is considered as significant and highly significant, respectively.

## Results

### Effect of K level on biomass, K concentration and accumulation of two wild barley accessions

A total of 99 barley accessions were evaluated in a previous experiment under low-K (0.01 mM) and normal K (1 mM) conditions [25]. XZ153 and XZ141 were identified as low-K tolerant and sensitive, respectively. Although two genotypes grew worse under low K than normal K (control), XZ153 was obviously less affected than XZ141 (Table 1). Hence, relatively dry weight of shoot (low K/control) was 90% for XZ153 and 64% for XZ141 (Table 1).

**Table 1:** K concentration and accumulation of two wild barley genotypes XZ153 (Low-K-tolerant) and XZ141 (Low-K-sensitive) under low and normal K levels.

Trait		Genotype					
		XZ153			XZ141		
		CK	LK	Relative	CK	LK	Relative
Dry weight (mg plant^−1^ DW)	Root	63.17a	60.33a	0.96	61.33a	46.17b	0.75
	Shoot	207.67a	187.67a	0.90	212.33a	135.50b	0.64
	Total	270.83a	248.00b	0.92	273.67a	181.67c	0.66
K concentration (mg g^−1^ DW)	Root	65.44a	10.08b	0.15	64.21a	9.70b	0.15
	Shoot	82.33a	31.08b	0.38	81.31a	26.15c	0.32
K accumulation (mg plant^−1^ DW)	Root	4.13a	0.61b	0.15	3.94a	0.45b	0.11
	Shoot	17.10a	5.83b	0.34	17.27a	3.54c	0.21
	Total	21.23a	6.44b	0.30	21.21a	3.99c	0.19

CK: Normal K level (1 mM K); LK: Low K level (0.01 mM K); Relative: LK/CK. For each line, different lowercase letters indicate significant differences (P<0.01) among the treatments and genotypes, n = 3.

Furthermore, the two wild barley accessions differed greatly in K concentration and accumulation (Table 1). There was little difference in both root and shoot concentrations between XZ153 and XZ141 under normal K. However, shoot K concentration of XZ153 was significantly higher than that of XZ141 under low-K (Table 1). K accumulation is a function of plant dry weight and K concentration. As a result, K accumulation of XZ153 was 1.61 times larger than that of XZ141 under low-K (Table 1).

### Evaluation of RNA-Seq reads and mapping results

In order to determine suitable time of sampling for RNA-Seq analysis, relative expression of *HvHAK1* at 6 h, 12 h, 24 h, 48 h, 5 d and 7 d after low-K treatment was compared (Figure S1). The results showed that the *HvHAK1* gene was up-regulated at 6 h after low-K stress, then remained little change at 12 h and 24 h. Obviously, roots has already sensed low-K signal and activated relevant signal transduction at 6 h after treatment, resulting in differential expression of the genes so as to cope with low-K stress. Interestingly, the expression level of the *HvHAK1* gene was significantly increased at 48 h in comparison with those at 6 h, 12 h or 24 h, and thereafter, remained little change at 5 d and 7 d (Figure S1). Thus, we took the samples at 6 h and 48 h for RNA-Seq analysis.

To obtain an overview of the transcriptome profiling of the early response to low-K stress in the two wild barley accessions, six sequencing cDNA libraries were constructed, and paired-end reads were sequenced using the Illumina platform. We got raw reads with length ranging from lower than 50 bp to as high as 101 bp. A total of 223 112 382 clean reads were generated by sequencing 6 cDNA libraries. All reads were categorized into three classes, including unmapped, multiple mapped and unique mapped reads. Of the 29–41 million clean reads from each library, 77–81% was mapped to unique locations, whereas 6–10% was mapped to multiple locations in the genome (Table S1). Meanwhile, the number of expressing genes found in each sample ranged from 54 322 to 57 516, thus providing massive data for further expression profiling analysis.

### Identification of differentially expressed genes (DEGs) and cluster analysis

**Figure 1:**
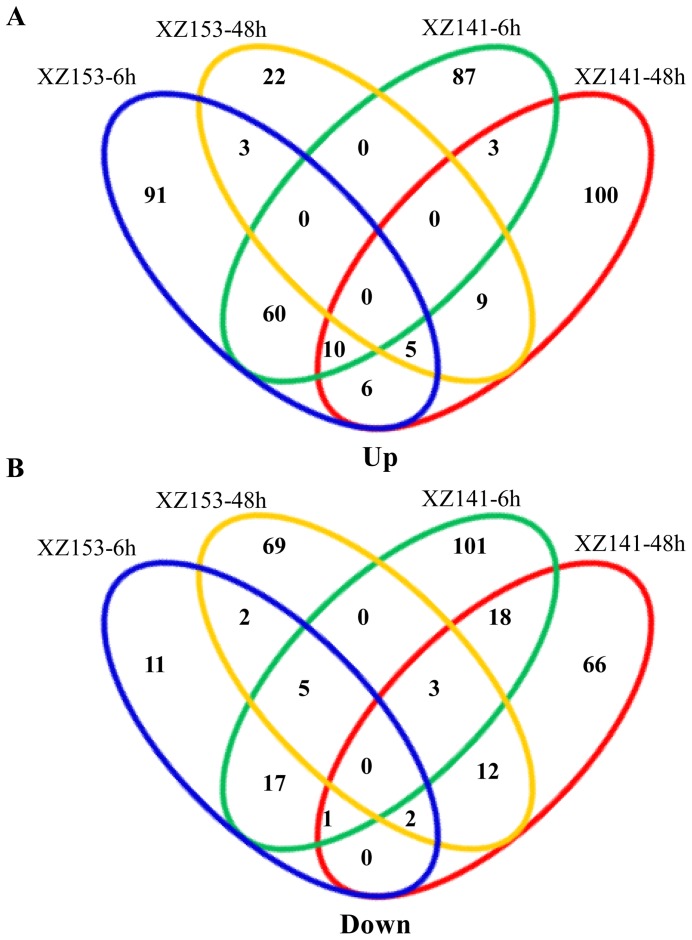
A Venn diagram describing overlaps among differentially expressed genes (DEGs) in XZ153 and XZ141. (A) Up-regulated genes at 6 h and 48 h after low-K treatment. (B) Down-regulated genes at 6 h and 48 h after low-K treatment.

Gene profiles of wild barley roots under both normal and low-K conditions were analyzed. FPKM (Fragments per kilo-base of exon per million fragments mapped reads) method was employed to normalize gene expression counts for the sequence. Previous studies suggested that sequencing with low FPKM may not provide reliable expression data statistically [28]. To minimize false positives, FPKM≥2 was required at least for one of the samples [28]. Additionally, differentially expressed genes were identified according to fold change and P value [32]. Hence, in this study, we set a screening threshold (FPKM≥2 at least in one of the samples, 2-fold change, P≤0.01) of differentially expressed genes. We used the same criteria for both XZ153 and XZ141 to obtain genes that had a significant response to low-K stress. A total of 692 genes showed differential expression at 6 h and 48 h under low-K stress in the two accessions (Table S2, Table S3). XZ141 had more differentially expressed genes than XZ153 (Figure 1). There were only 137 differentially expressed genes, which were commonly found in both XZ153 and XZ141. There were more DEGs at 6 h than at 48 h in the two accessions (Table S4, Table S5). Nearly same amount of DEGs were up-regulated and down-regulated at 6 h and 48 h in XZ141 (Figure 1). However, the gene expression pattern in XZ153 differed from that in XZ141. The number of up-regulated genes in XZ153 was almost four times as large as that of down-regulated ones at 6 h (Figure 1).

Meanwhile, the 692 DEGs were divided into eight groups, based on their expression pattern by cluster analysis (Genesis 1.7.5) (Figure S2). Clusters 1 and 2 included the genes with continuous positive or negative response along the whole time course; clusters 3 and 4 included the genes with persistent positive or negative response; clusters 5 and 6 included the genes with latent positive or negative response, and cluster 7 and 8 referred to the genes with initial positive or negative response (Figure S2). In view of the differentially expressed gene patterns and DEGs, it can be suggested that XZ153 has a distinct mechanism differing from XZ141 in response to low-K stress. Thus, it is valuable to make further analysis of different responses to low-K stress between the two accessions.

**Figure 2:**
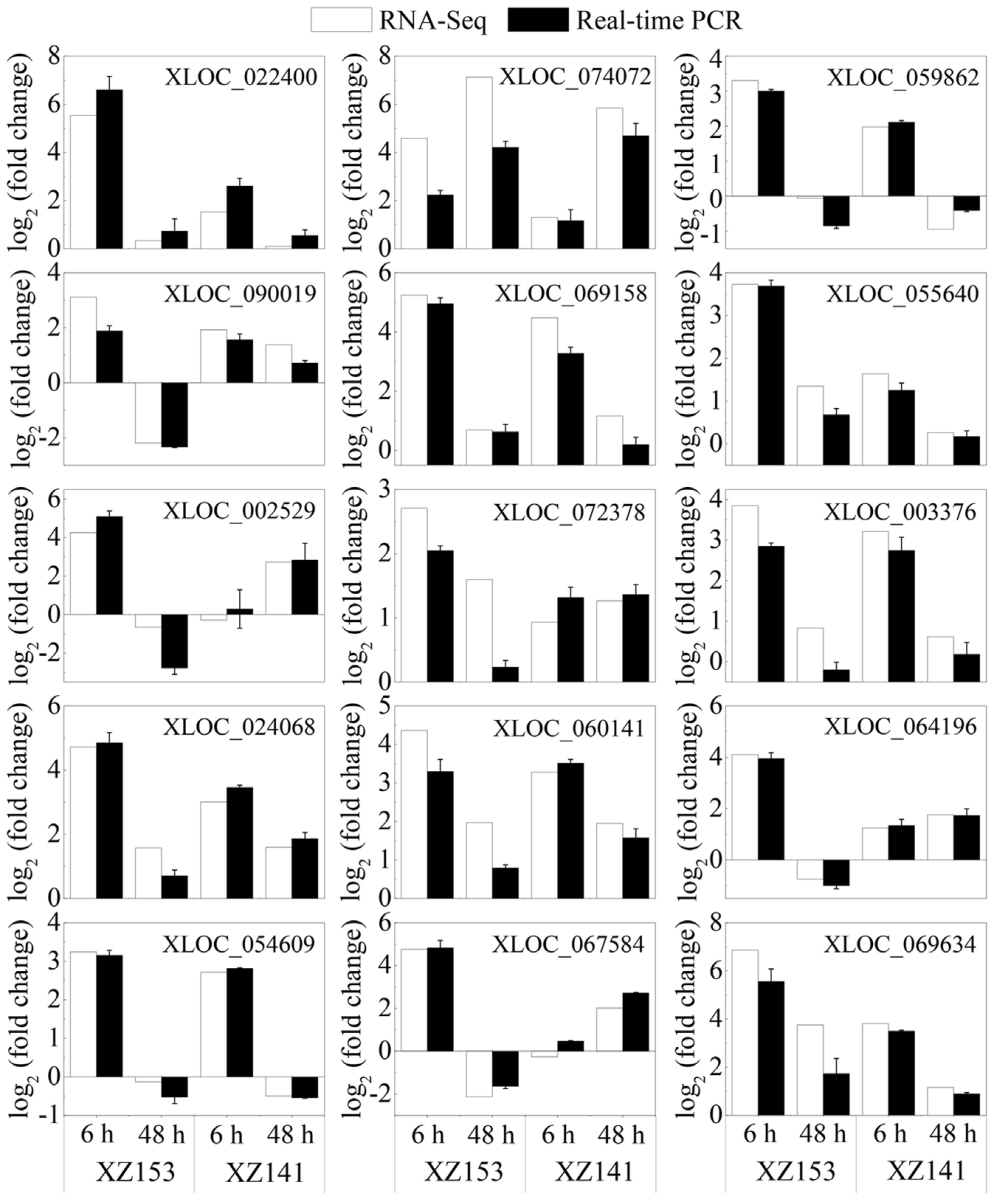
Quantitative real-time PCR validation of 15 differentially expressed genes. Gene-specific primers used for real-time PCR are listed in Table S6.

To assess the validity of the RNA-Seq data, 15 DEGs were randomly selected for real-time PCR analysis (Table S6) and they included CBF protein 4 (XLOC_069634), transporter HKT7-like (XLOC_074072), late embryogenesis abundant protein(XLOC_022400), dehydrin 1 (XLOC_090019), kelch repeat-containing F-box family protein (XLOC_069158), chaperone protein DnaJ (XLOC_024068), Ring-h2 finger protein ATL32-like (XLOC_003376), low temperature and salt responsive protein family (XLOC_054609), three pathogenesis-related proteins (XLOC_002529, XLOC_064196, XLOC_067584), response regulator like protein (XLOC_072378), phosphomethylpyrimidine synthase (XLOC_059862) and unknown proteins (XLOC_060141, XLOC_055640) (Table S6). For most of these genes, their expression patterns of the real-time PCR were highly consistent with those shown in the RNA-Seq data (Figure 2).

### Transcription factors (TFs) in the differentially expressed genes (DEGs)

**Figure 3:**
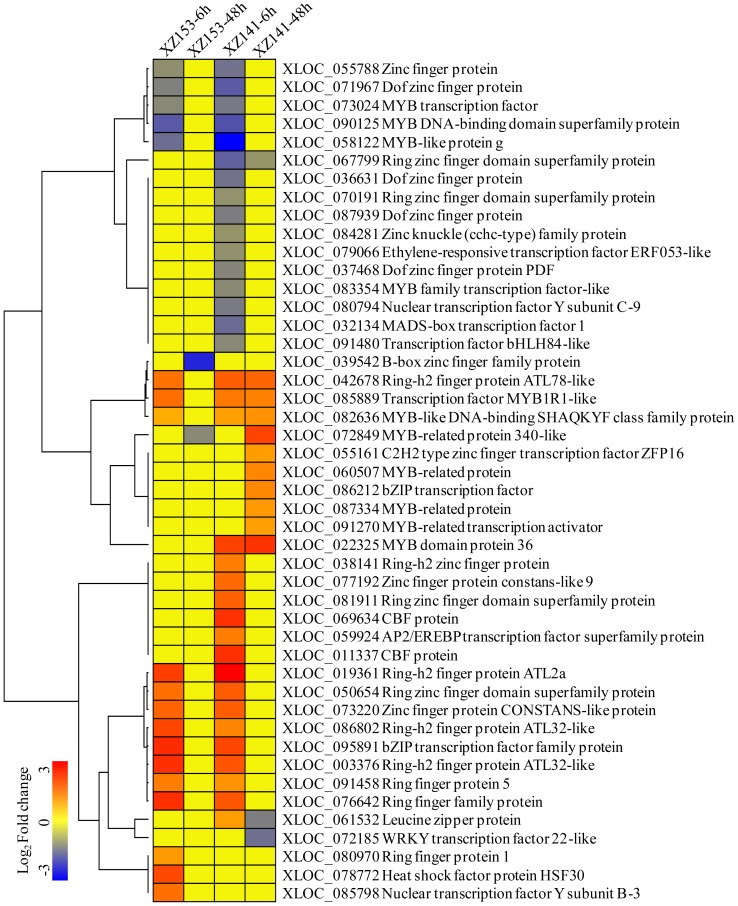
Average linkage hierarchical cluster analysis of Transcription factors (TFs) identified in differentially expressed genes (DEGs). The sample and treatments are displayed above each column. Genes are displayed by different colors. Relative levels of expression are showed by a color gradient from low (blue) to high (red).

Transcription factors are essential for regulation of gene expression. In this study, 46 DEGs encoding transcription factors were identified, and they belonged to diverse families, such as Zinc finger (22), MYB (11), bZIP (3), CBF (2), NF-Y (2), ERF (1), WRKY (1), bHLH (1), MADS-box (1), AP2/EREBP (1), HSF (1) (Figure 3). Interestingly, we found that the proteins with zinc finger domains were the most enriched among the TFs, accounting for 48% of all DEGs (Figure 3). According to the expression patterns, all the TFs could be clustered into several categories. Overall, most TFs in both two accessions displayed a short-term response, and then returned to the original expression level, although a few of them were only differentially expressed at 48 h (Figure 3). Hence, we were focused on these TFs which were significantly up-regulated in XZ153, but remained little change in XZ141, or remained little change in XZ153 but down-regulated in XZ141.

### Transporter and kinase

In the current study, six DEGs encoding K transporters were detected. Surprisingly, expression level of the genes encoding K transporters was not changed obviously until after 48 h stress in XZ141 (Table 2). By contrast, three of them (XLOC_030313, XLOC_086787, XLOC_074072) were up-regulated at 6 h in XZ153, including a HKT7-like gene with higher expression (Table 2). Meanwhile we also found that the expression of the genes encoding nitrate and ammonium transporters changed greatly under low-K stress (Table 2), indicating that K and N uptake is under cross-talking regulation. In addition, the genes associated with ion uptake or translocation were discovered, which is probably involved in ion homeostasis under low-K stress (Table 2). Moreover, some DEGs encoding protein kinases of different groups were also identified (Table 2). Among these genes, there were 4 CBL-interacting protein kinases, 3 leucine-rich repeat receptor-like protein kinases and 2 cysteine-rich receptor-like protein kinases. On the whole, the expression of those groups was mostly down-regulated in XZ141, but up-regulated or unchanged in XZ153.

**Table 2:** Genes encoding protein transporters and kinases showing genotypic difference expression in response to low K stress.

Group	Gene id	Log2(Fold change)				Seq description
		XZ153		XZ141		
		6 h	48 h	6 h	48 h	
Potassium	XLOC_030313	2.62	4.38		4.59	High-affinity potassium transporter
	XLOC_086787	2.36	2.82		3.06	High-affinity potassium transporter
	XLOC_032661		2.01		2.89	High-affinity potassium transporter
	XLOC_033262		1.89			Potassium transporter
	XLOC_035041				3.05	High-affinity potassium transporter
	XLOC_074072	4.59	7.13		5.84	Transporter HKT7-like
Nitrate	XLOC_031163	2.14				Probable nitrate transporter
	XLOC_093217	2.04			2.22	Nitrate transporter -like
	XLOC_082123		−2.61	−1.89	−3.79	Nitrate transporter -like
	XLOC_020339				1.94	High-affinity nitrate transporter -like
Ammonium	XLOC_050205				2.83	Ammonium transporter
	XLOC_027452				1.86	Ammonium transporter
Yellow-strike	XLOC_082435	−3.11	−4.61	−5.10		Metal-nicotianamine transporter
	XLOC_082568	−3.19	−4.05	−4.89		Metal-nicotianamine transporter
	XLOC_082319	−3.19	−4.62	−5.00		Metal-nicotianamine transporter
	XLOC_090161			−2.26		Probable metal-nicotianamine transporter
Mate	XLOC_071812	2.76			2.37	MATE efflux family protein
	XLOC_079551	2.34				MATE efflux family protein
	XLOC_079205				−2.23	MATE efflux family protein, expressed
	XLOC_035193			−4.33	−2.32	MATE efflux family protein
CIPK	XLOC_095894	4.59		4.03		CBL-interacting protein kinase
	XLOC_050803			−2.04		CBL-interacting protein kinase
	XLOC_083910			−2.37		CBL-interacting protein kinase
	XLOC_065318			−2.56		CBL-interacting protein kinase
LRR	XLOC_066629				−3.00	LRR receptor-like protein kinase
	XLOC_081108			3.16		LRR receptor-like protein kinase
	XLOC_087240	2.80				LRR receptor-like protein kinase
	XLOC_084744			−2.61		LRR receptor-like protein kinase
CRK	XLOC_007825			−3.52	−3.99	Cysteine-rich receptor-like protein kinase
	XLOC_071068	5.55				Cysteine-rich receptor-like protein kinase

### DEGs related to hormone signaling and oxidative stress

Heatmap clustering analysis was performed to study differentially expressed genes involved in hormone signaling in the two barley accessions under low-K stress (Figure S3). A total of 24 hormone-related DEGs were found, including gibberellin (4), jasmonate (5), cytokinin (3), auxin/IAA (8), ethylene (1) and abscisic (3) (Figure S3). Meanwhile, the key enzymes, such as peroxidase (6), cytochrome P450 (8) and glutathione S-transferase (8) were indentified, and they are critical for the regulation of ROS production and reducing cellular damage in the response to low-K stress (Figure S4). At 6 h after low-K treatment, there were few oxidative stress-related genes (Figure S4). However, the changed genes increased dramatically at 48 h after treatment. Moreover, there were more differentially expressed genes in XZ141 than in XZ153 (Figure S4).

### Gene ontology (GO) function and KEGG analysis of low-K tolerance related DEGs

**Figure 4:**
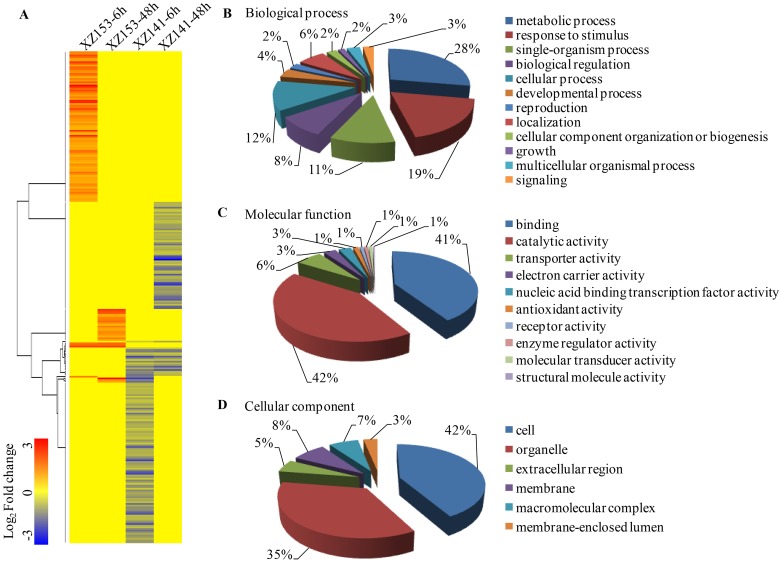
Hierarchical cluster and gene ontology (GO) categories analysis of low-K tolerance related DEGs. A total of 294 low-K tolerance related DEGs were performed on (A) Hierarchical cluster analysis. The samples and treatments are displayed above each column. Genes are displayed by different colors and relative levels of expression are showed by a color gradient from low (blue) to high (red). Gene ontology categories from three levels: (B) Molecular function; (C) Biological process; (D) Cellular component.

We are focused on those DEGs, whose expression was significantly up-regulated in XZ153 roots, but down-regulated/unchanged in XZ141, or remained little changed in XZ153 but down-regulated in XZ141. In this study, a total of 294 DEGs met the above criteria and were further investigated. Hierarchical clustering analysis of those DEGs was performed and they could be mainly grouped into four classes (Figure 4A). GO functional enrichment analysis revealed that the genes associated with binding (GO: 0005488), catalytic activity (GO: 0003824) and transporter activity (GO: 0005215) were significantly enriched, accounting for as much as 89% of molecular function (Figure 4B). The processes represented by the GO terms ‘metabolic process', ‘response to stimulus’, ‘single-organism process’, ‘biological regulation’ and ‘cellular process’, accounted for the majority of the biological process (Figure 4C). Meanwhile, DEGs related to low-K tolerance also acted as diverse cellular components (Figure 4D).

**Figure 5:**
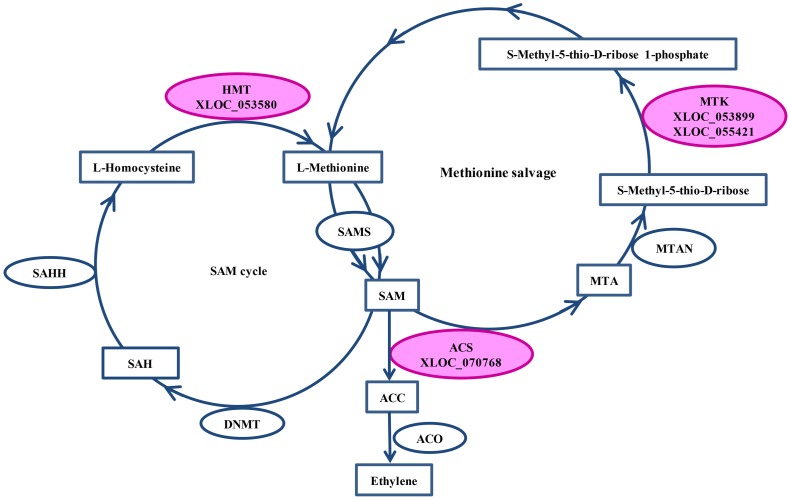
DEGs were mapped to SAM cycle and methionine salvage involving in ethylene biosynthesis process. The three differentially expressed enzymes were colored in pink. SAM: S-Adenosyl-L-methionine; SAMS: S-Adenosyl-L-methionine synthase; ACC: S-Aminocyclopropane-1-carboxylate; ACS: S-Aminocyclopropane-1-carboxylate synthase; ACO: S-Aminocyclopropane-1-carboxylate oxidase; MTA: S-Methyl-5′-thioadenosine; MTAN: 5′-Methylthioadenosine Nucleosidase; MTK: S-methyl-5-thioribose kinase; SAH: S-Adenosyl-L-homocysteine; SAHH: S-Adenosyl-L-homocysteine hydrolase; DNMT: DNA (cytosine-5-)-methyltransferase; HMT: Homocysteine S-methyltransferase.

Totally, 294 DEGs encoding various enzymes were further investigated for KEGG pathway enrichment. Forty-seven enzymes were assigned to 32 KEGG pathways, including amino acid, nucleotide, lipid, carbohydrate, energy and other metabolisms (Figure S5). Based on FPKM value, we found 6 enzymes, which were involved in starch and sucrose metabolisms, and altered markedly (Figure S6). In addition, six pathways, classified as lipid metabolism, including alpha-linolenic acid, fatty acid biosynthesis, glycerolipid, glycerophospholipid, linoleic acid and sphingolipid metabolisms, differed in expression patterns between XZ153 and XZ141 (Figure S5). Among them, two DEGs encoding lipase (EC: 3.1.1.3) and 13S-lipoxygenase (EC: 3.2.1.23) showed up-regulation in XZ153, but unchanged in XZ141; whereas three enzymes (EC: 3.2.1.23, EC: 6.3.4.14, EC: 2.3.1.15) were unchanged in XZ153, and down-regulated in XZ141. Meanwhile, the expression of two key enzymes (Homocysteine S-methyltransferase and S-methyl-5-thioribose kinase) participating in S-Adenosyl-L-methionine (SAM) cycle and methionine salvage process, was unchanged in XZ153, but down-regulated in XZ141 (Table S7). Furthermore, one DEGs encoding S-Aminocyclopropane-1-carboxylate synthase (ACS), a key rate-limiting enzyme in ethylene biosynthesis pathway, was also indentified, which showed normal expression in XZ153, and down-regulation in XZ141 (Figure 5, Table S7).

## Discussion

Potassium is an essential macronutrient for plant growth and development. However, K deficiency in soil is quite common and becomes more severe in crop production [7]. The most effective approach of overcoming K deficiency is to develop the crop cultivars with high tolerance to low-K stress or high K use efficiency. In the present study, we used the RNA-Seq to reveal the transcriptome profiling of two wild barley accessions differing in low K tolerance. Firstly, phenotypic responses of these two genotypes were compared. It was reported by Hermans *et al* (2006) that biomass was the final result of plant growth and development, so relatively dry weight was often used as indicators of plant tolerance to low nutrition stress [33], [34]. Under low K, root and shoot growth was dramatically inhibited for XZ141, whereas remained less effect for XZ153 (Table 1), proving that XZ153 is more tolerant to low-K stress than XZ141. Meanwhile, the reduction of K concentration and accumulation in the shoot under low K also differed greatly between the two genotypes, XZ153 being less reduced than XZ141, indicating that XZ153 had the higher capability of K absorption and translocation.

The capacity of K absorption and translocation is related to K transporters and channels, which belong to high- and low-affinity uptake system, respectively. A number of K transporters and channels have been functionally characterized in plants [3], [35], [36]. It has been demonstrated that *HvHAK1*, as well as *AtHAK5* and *OsHAK1* are all assigned to the KT/KUP/HAK family and could be induced by low external potassium [37]–[41]. The previous studies reported that there were seven HAK-type transporters in the response to low-K stress in rice; including up- and down-regulated expression patterns [42]. However, in this study, all the HAK transporter proteins were up-regulated in the two genotypes. Interestingly, some of them were up-regulated in XZ153 as early as at 6 h after treatment, but not in XZ141 at that time (Table 2). Thus, it may be assumed that a rapid response to low K stress happened in XZ153 is attributed to its more K uptake and accumulation in plants, resulting in higher low K tolerance.

Transcription factors are a kind of proteins that are bound to *cis*-regulatory elements and can regulate gene expression [43]. The roles of Zinc finger protein and some members of the MYB transcription factor family in abiotic stress tolerance have been well documented [44]–[47]. In the present study, 48 DEGs encoding transcription factors were characterized (Figure 3), with Zinc finger and MYB being the largest components, similar to the results that were obtained in rice plants exposed to K starvation [48]. Obviously, some members of those two families are associated with the responses to low-K stress, at least in rice and barley. Nevertheless, nuclear factor Y (NF-Y), a conserved heterotrimeric and CCAAT-specific domain, composed of NF-YA, NF-YB, NF-YC, was only observed in this study, and was not found in rice and soybean subjected to low-K stress [42], [49]. Although the members of NF-Y are involved in multiple biological functions, its relation with low-K stress tolerance has been not reported up to data [50], [51]. Meanwhile, many TFs, mainly involved in plant growth and development, were repressed in XZ141. It may be assumed that more growth inhibition of XZ141 is described to lower K concentration in plant tissues under low-K stress.

The plant hormone, ethylene is involved in many aspects of the plant life cycle and its production is tightly regulated in response to environmental stimuli from both of biotic and abiotic stresses [52]. Hence, the mechanisms related to biosynthesis of ethylene have been intensively studied. Using chemical and genetic approaches, Jung *et al*., (2009) found the ethylene-ROS pathway in *Arabidopsis* that ethylene stimulated the production of ROS and played an important role in low-K stress [53]. As S-Aminocyclopropane-1-carboxylate (ACC) is a direct precursor of ethylene biosynthesis, the step from the (S-Adenosyl-L-methionine synthase) SAM to ACC, catalyzed by ACC synthase (ACS) and usually considered as a rate-limiting step in ethylene synthesis, is very important in this process. In addition to the two enzymes involved in SAM cycle and methionine salvage, a key enzyme ACS was also found in the current study (Figure 5). In particular, the DEGs encoding these three enzymes were inhibited in sensitive genotype XZ141 under low-K stress, but unchanged in the tolerant genotype XZ153 (Table S7). Meanwhile, an ethylene-responsive transcription factor ERF053-like was down-regulated simultaneously in XZ141, while little change in XZ153 (Figure 3). The AP2/ERF transcription factor, RAP2.11 in *Arabidopsis*, is a factor in regulating transporter through biding to promoter of *AtHAK5* and has been also identified as a component of ethylene-ROS pathway under low-K stress [54]. Obviously, the ethylene response pathway may account for the genotypic difference in low-K tolerance, which may be reflected by K uptake capability.

KEGG is a major public pathway-related database, which is valuable for research on the complex biological behaviors of genes. In the present study, 294 DEGs should be specially addressed in view of their expression patterns, i.e. the genes being up-regulated in tolerant genotype XZ153 and remained unchanged or down-regulated in sensitive genotypes XZ141, or unchanged in XZ153, and down-regulated in XZ141 (Figure 4A). KEGG metabolic pathway enrichment of these genes will likely make insight into the mechanism of low-K tolerance. The difference was found in the regulation of some metabolisms between the two genotypes, including carbohydrate, cysteine, methionine and lipid metabolisms (Figure S5). Armengaud *et al* (2009) reported that the genes involved in carbohydrate metabolism played a vital role in the regulation of potassium nutrition in plants [55]. In this context, these differences in metabolic pathways may lead to different energy distribution and capacity of adaptation to low-K stress in the two genotypes.

**Figure 6:**
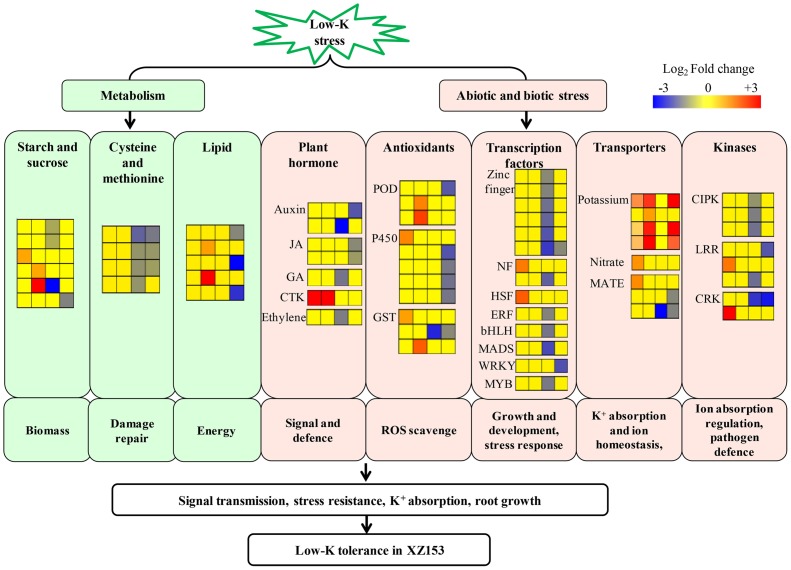
A hypothetical model of low-K tolerance mechanism underlying in XZ153. Genes are shown by different colors and relative expression levels are shown by a color gradient from low (blue) to high (red). For each heatmap from left to right: XZ153-6h (first column), XZ153-48h (second column), XZ141-6h (third column), XZ141-48h (fourth column).

In conclusion, we applied high-throughput Next Generation Sequencing (NGS) technique to investigate gene expression profiling of two Tibetan wild barley genotypes in response to low-K stress. The results demonstrate that the response of XZ153 and XZ141 to low-K stress differed dramatically in the transcriptional level. Despite the complexity of responses to low-K, a hypothetical model could be suggested for low-K tolerance mechanism in XZ153, based on the available results (Figure 6). While the difference in K absorption and accumulation in plant tissues between the two genotypes may explain the different growth inhibition under low K stress. In our understanding, this is a first comprehensive study of low K tolerance in Tibetan wild barley at transcriptome level. In addition, the current results provide some candidate genes, which can be used in barley breeding for improving low-K tolerance.

## Supporting Information

Figure S1 **Real-time PCR analysis of the** ***HvHAK1*** **gene under low K treatment.** * represents significant difference according to the Duncan's multiple range, P<0.05, n = 3. Primers of *HvHAK1* and GAPDH for RT-PCR are listed in Table S6.
(PDF)

Figure S2 **Cluster analysis of the DEGs in the two genotypes.** Y-axis represents the gene expression values (FPKM) transformed by logarithms, base 2. The middle white line indicates the gene expression trend of each cluster.
(PDF)

Figure S3 **Heat Map analysis of DEGs involved in hormone signaling in XZ153 and XZ141.** The sample and treatments are displayed above each column. Genes are displayed by different colors. Relative levels of expression are showed by a color gradient from low (blue) to high (red).
(PDF)

Figure S4 **Heat Map analysis of DEGs involved in oxidative stress-related in XZ153 and XZ141.** The samples and treatments are displayed above each column. Genes are displayed by different colors. Relative levels of expression are showed by a color gradient from low (blue) to high (red).
(PDF)

Figure S5 **KEGG overview of low-K tolerance related DEGs under low K stress.** X-axis represents the number of enzymes participating in each pathway; Y- axis depicts the different pathway.
(PDF)

Figure S6 **DEGs related to low-K tolerance were mapped to the starch and sucrose metabolism pathway.** The column marked in color indicates the differentially expressed enzymes. Four small squares from left to right represent different treatment time of the two varieties (XZ153-6h, XZ153-48h, XZ141-6h, XZ141-48h). Gene expression is displayed by different colors and the relative levels of expression are showed by a color gradient from low (blue) to high (red).
(PDF)

Table S1 **Summary of RNA-seq data and mapping results.**
(DOC)

Table S2 **The FPKM value of 692 DEGs.**
(XLS)

Table S3 **Gene accession numbers and sequences of 692 DEGs.**
(XLS)

Table S4 **DEGs at 6 h and 48 h after low K treatment in XZ153.**
(XLS)

Table S5 **DEGs at 6 h and 48 h after low K treatment in XZ141.**
(XLS)

Table S6 **The primers used in real-time PCR.**
(DOC)

Table S7 **DEGs are involved in Starch and Sucrose metabolism or Cysteine and Methionine metabolism.**
(DOC)
