# Supplementary material for: Comparative Transcriptome Profiling of Two Tibetan Wild Barley Genotypes in Responses to Low Potassium
Source: PLoS One. 2014 Jun 20;9(6):e100567. doi: 10.1371/journal.pone.0100567 (PMC4065039; doi:10.1371/journal.pone.0100567)
Supplement: Figure S1 — Real-time PCR analysis of the HvHAK1 gene under low K treatment. * represents significant difference according to the Duncan's multiple range, P<0.05, n = 3. Primers of HvHAK1 and GAPDH for RT-PCR are listed in Table S6. (PDF) [file pone.0100567.s001.pdf]

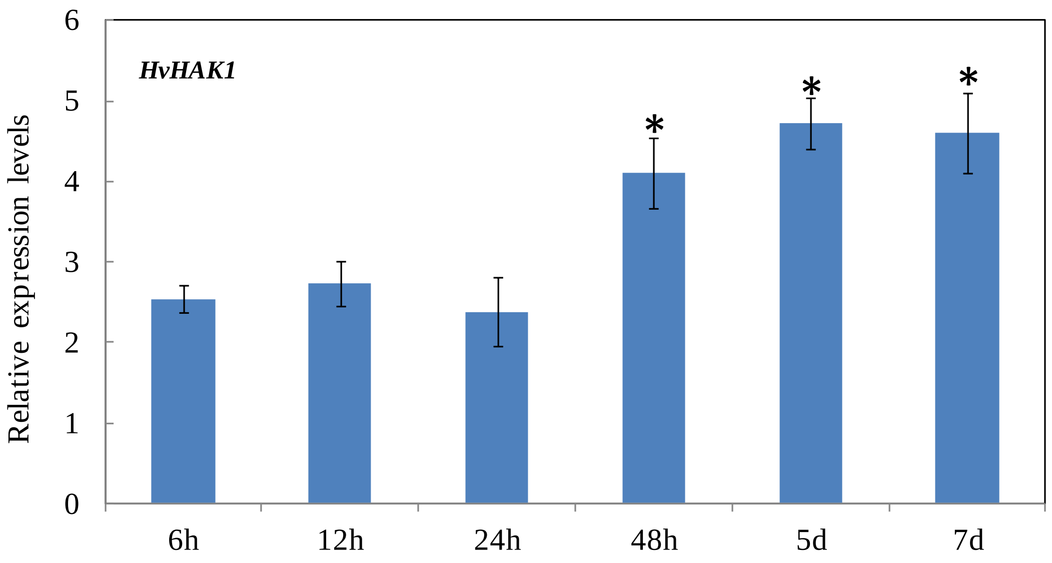

Figure S1 Real-time PCR analysis of the *HvHAK1* gene under low K treatment. * Means significantly different according to the Duncan's multiple range, $P<0.05$, $n = 6$. Primers of *HvHAK1* and GAPDH for RT-PCR are listed in Table S6.
