# Supplementary material for: Comparative Transcriptome Profiling of Two Tibetan Wild Barley Genotypes in Responses to Low Potassium
Source: PLoS One. 2014 Jun 20;9(6):e100567. doi: 10.1371/journal.pone.0100567 (PMC4065039; doi:10.1371/journal.pone.0100567)
Supplement: Figure S2 — Cluster analysis of the DEGs in the two genotypes. Y-axis represents the gene expression values (FPKM) transformed by logarithms, base 2. The middle white line indicates the gene expression trend of each cluster. (PDF) [file pone.0100567.s002.pdf]

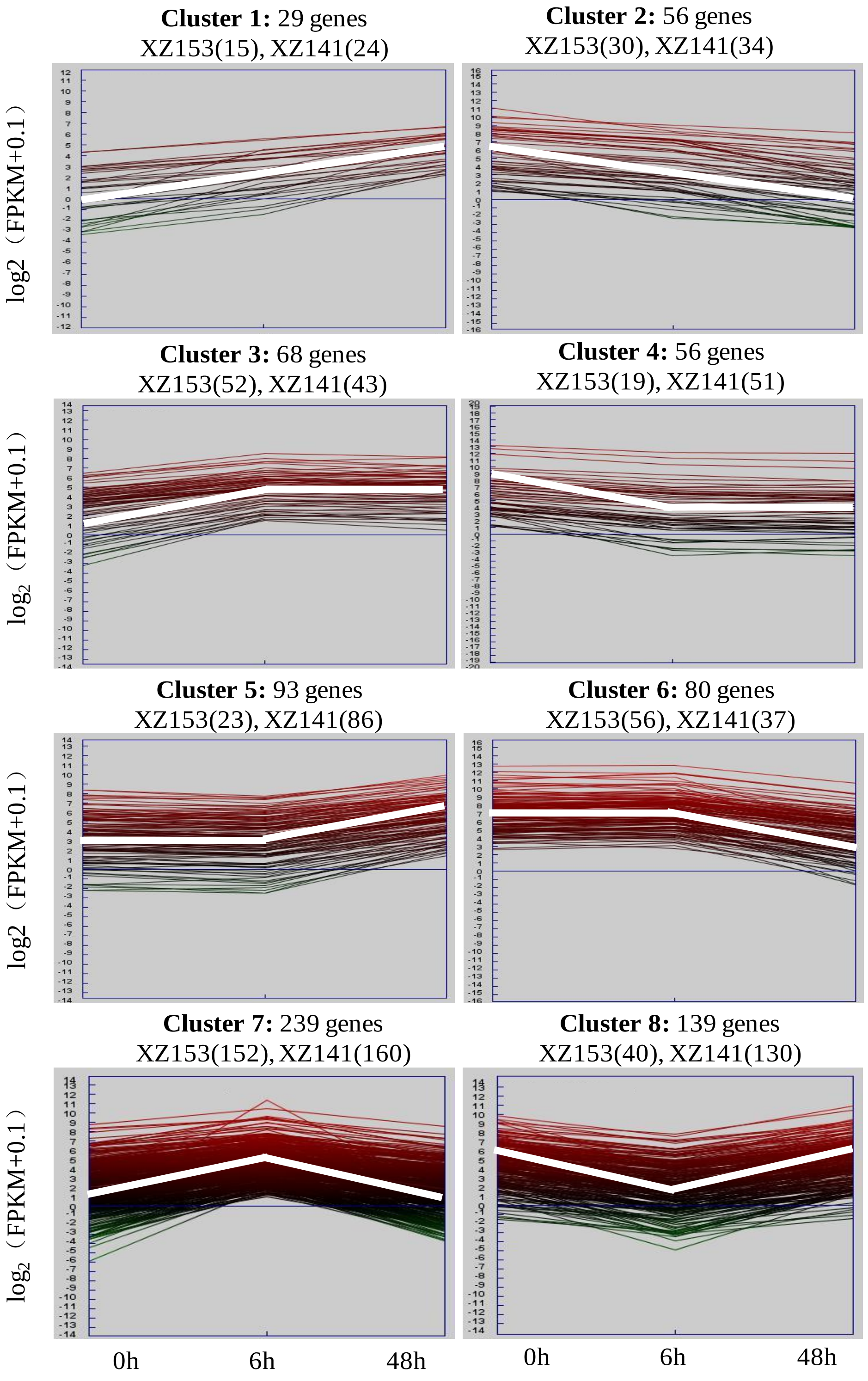

Figure S2. Cluster analysis of the DEGs in the two genotypes. 692 DEGs at 6 h and 48 h after low K treatment were clustered on the basis of the k-means method. Y-axis represents the gene expression values (FPKM) transformed by logarithms, base 2. The middle white line indicates the gene expression trend of each cluster.
