# Supplementary material for: Comparative Transcriptome Profiling of Two Tibetan Wild Barley Genotypes in Responses to Low Potassium
Source: PLoS One. 2014 Jun 20;9(6):e100567. doi: 10.1371/journal.pone.0100567 (PMC4065039; doi:10.1371/journal.pone.0100567)
Supplement: Figure S3 — Heat Map analysis of DEGs involved in hormone signaling in XZ153 and XZ141. The sample and treatments are displayed above each column. Genes are displayed by different colors. Relative levels of expression are showed by a color gradient from low (blue) to high (red). (PDF) [file pone.0100567.s003.pdf]

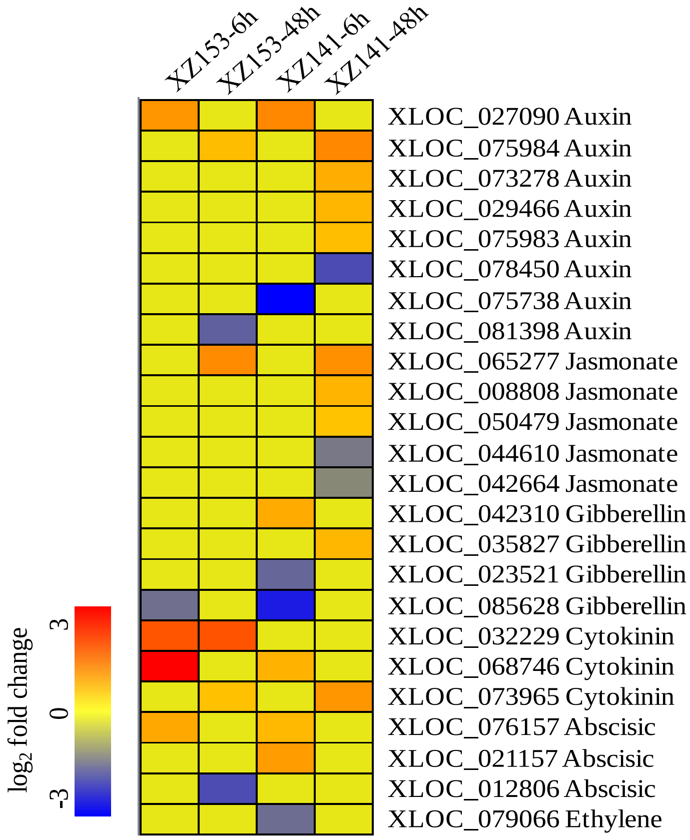

Figure S3. Heat Map analysis of DEGs involved in hormone signaling in XZ153 and XZ141. The sample and treatments are displayed above each column. Genes are displayed by different colors. Relative levels of expression are showed by a color gradient from low (blue) to high (red).
