# Supplementary material for: Comparative Transcriptome Profiling of Two Tibetan Wild Barley Genotypes in Responses to Low Potassium
Source: PLoS One. 2014 Jun 20;9(6):e100567. doi: 10.1371/journal.pone.0100567 (PMC4065039; doi:10.1371/journal.pone.0100567)
Supplement: Figure S4 — Heat Map analysis of DEGs involved in oxidative stress-related in XZ153 and XZ141. The samples and treatments are displayed above each column. Genes are displayed by different colors. Relative levels of expression are showed by a color gradient from low (blue) to high (red). (PDF) [file pone.0100567.s004.pdf]

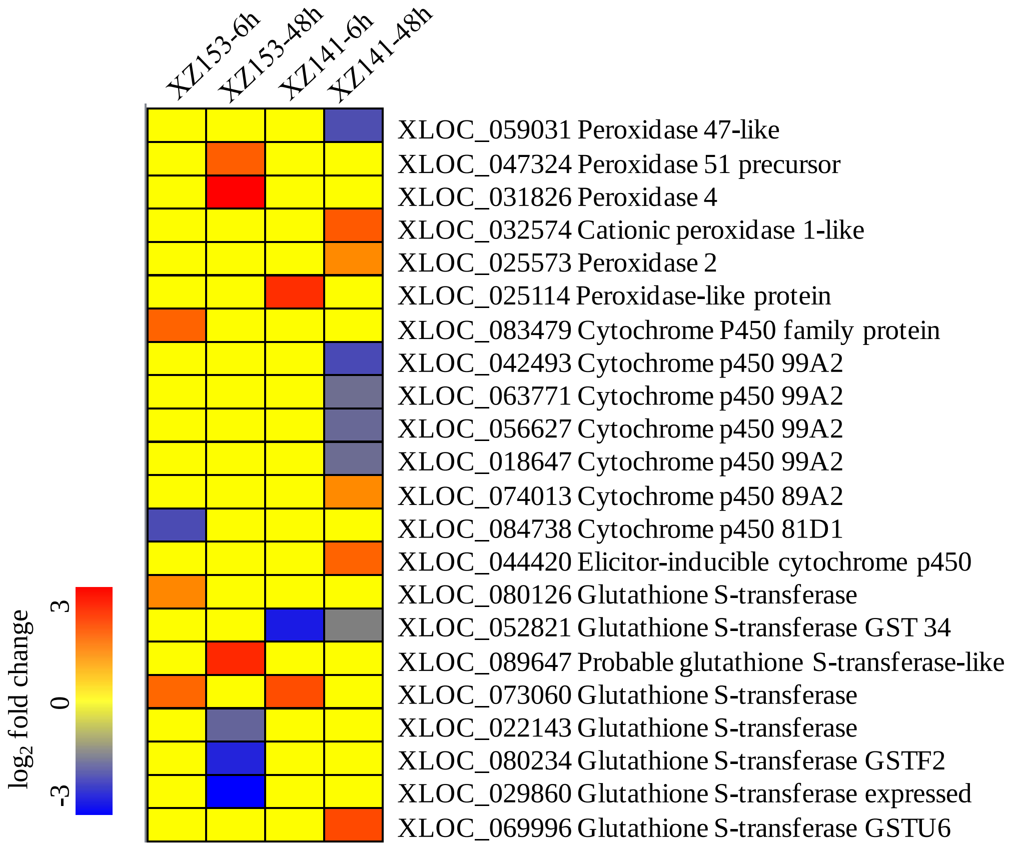

Figure S4. Heat Map analysis of DEGs involved in oxidative stress-related in XZ153 and XZ141. The sample and treatments are displayed above each column. Genes are displayed by different colors. Relative levels of expression are showed by a color gradient from low (blue) to high (red).
