# Supplementary material for: Comparative Transcriptome Profiling of Two Tibetan Wild Barley Genotypes in Responses to Low Potassium
Source: PLoS One. 2014 Jun 20;9(6):e100567. doi: 10.1371/journal.pone.0100567 (PMC4065039; doi:10.1371/journal.pone.0100567)
Supplement: Figure S5 — KEGG overview of low-K tolerance related DEGs under low K stress. X-axis represents the number of enzymes participating in each pathway; Y- axis depicts the different pathway. (PDF) [file pone.0100567.s005.pdf]

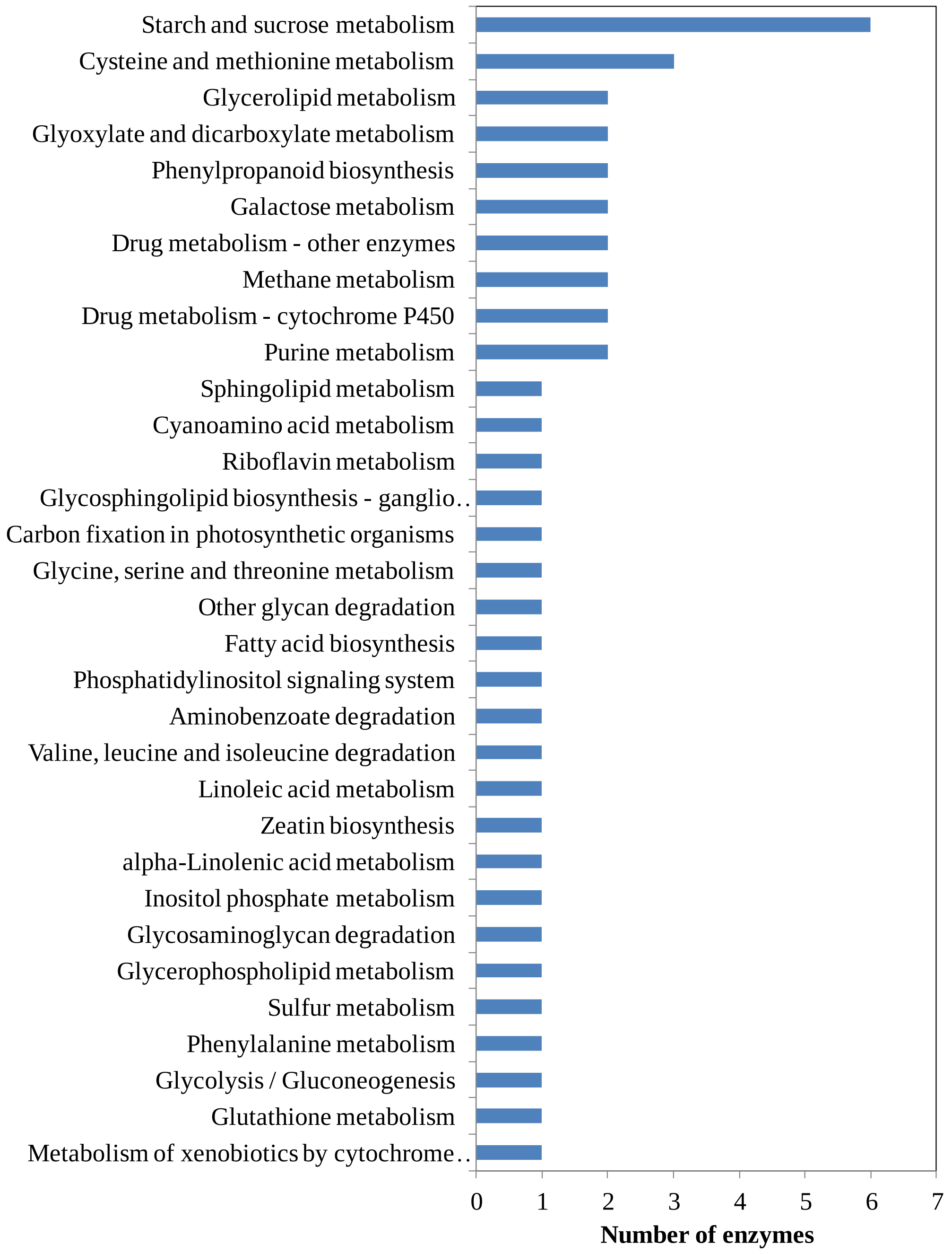

Figure S5. KEGG overview of low-K tolerance related DEGs under low K stress. X-axis represents the number of enzymes participating in each pathway; Y- axis depicts the different pathway.
