# Supplementary material for: Comparative Transcriptome Profiling of Two Tibetan Wild Barley Genotypes in Responses to Low Potassium
Source: PLoS One. 2014 Jun 20;9(6):e100567. doi: 10.1371/journal.pone.0100567 (PMC4065039; doi:10.1371/journal.pone.0100567)
Supplement: Table S1 — Summary of RNA-seq data and mapping results. (DOC) [file pone.0100567.s007.doc]

Table S1. Summary of RNA-seq and mapping results.

| Genotype |  | XZ153 | | |  | XZ141 | | |
| --- | --- | --- | --- | --- | --- | --- | --- | --- |
| Sample |  | 0h | 6h | 48h |  | 0h | 6h | 48h |
| Total Clean Reads |  | 41133320 | 40948778 | 38492262 |  | 28667730 | 36357330 | 37512962 |
| Unmapped reads |  | 5283636 | 5253904 | 4671341 |  | 4107207 | 6216104 | 5884669 |
| Multiple mapped reads |  | 3996411 | 2684994 | 2624647 |  | 1788899 | 2155502 | 2363395 |
| Uniquely mapped reads |  | 31848642 | 33003769 | 31190616 |  | 22767365 | 27980295 | 29259311 |
| Read-1 |  | 15897876 | 16729430 | 15827950 |  | 11548265 | 14200890 | 14839298 |
| Read-2 |  | 15950766 | 16274339 | 15362666 |  | 11219100 | 13779405 | 14420013 |
| Reads map to ‘+’ |  | 15941990 | 16496062 | 15590185 |  | 11373088 | 13980015 | 14624449 |
| Reads map to ‘-’ |  | 15906652 | 16507707 | 15600431 |  | 11394277 | 14000280 | 14634862 |
| Non-splice reads |  | 21548180 | 22669186 | 21618983 |  | 15835834 | 19343841 | 20854806 |
| Splice reads |  | 10300462 | 10334583 | 9571633 |  | 6931531 | 8636454 | 8404505 |
| Express gene number |  | 55157 | 57516 | 57028 |  | 55001 | 56435 | 57211 |
