# Supplementary material for: Comparative Transcriptome Profiling of Two Tibetan Wild Barley Genotypes in Responses to Low Potassium
Source: PLoS One. 2014 Jun 20;9(6):e100567. doi: 10.1371/journal.pone.0100567 (PMC4065039; doi:10.1371/journal.pone.0100567)
Supplement: Table S6 — The primers used in real-time PCR. (DOC) [file pone.0100567.s012.doc]

Table S6. Primers were listed for real-time PCR

| Gene ID | Description | Primer sequence (From 5′ to 3′) |
| --- | --- | --- |
| XLOC_022400 | Late embryogenesis abundant protein | F: AGCAAACCCATCAACCTAGC  R: GACCGATCTCGTGTGAGTTG |
| XLOC_074072 | Transporter HKT7-like | F: AGGTGATCATTGCAGGCACC  R: TGACACACTGAAGGCTCCCT |
| XLOC_054609 | Low temperature and salt responsive protein | F: CTGCAACAGTCTTGGAGGTG  R: CCAGAATGGTCAGCAAGAGA |
| XLOC_059862 | Phosphomethylpyrimidine synthase | F: CCAGCAAAACCCAGGCAATC  R: GCATTCTTTGGTGCTCCGTG |
| XLOC_090019 | Dehydrin 1 | F: ACTCGCAGCAGTTCAACACTT  R: GTACTCCATTGTCGCGCCTT |
| XLOC_069158 | Kelch repeat-containing F-box family protein | F: ATCTCCCACCTTCTTCTGCATC  R: ATCAAACATGCCTCGCTCAC |
| XLOC_060141 | Unknown protein | F:TCCCCCTCGGCATATAGCTT  R: TCTCGCAGCATCATCGTCAT |
| XLOC_064196 | Pathogenesis-related protein 1 | F: CACGTCATCGTCTCTCGTCC  R: TTACTCGCTCGGTCCCTCTA |
| XLOC_072378 | Response regulator like protein | F: AGCTTCCAATCCAGCGATCC  R: CGCAGATCAAGTAGCCACCA |
| XLOC_003376 | Ring-h2 finger protein ATL32-like | F: CTCACAAGTCGCTGGTCACA  R: ACGACGGCATAGTTGGTTGA |
| XLOC_024068 | Chaperone protein DnaJ | F: AACTCGTGTCGCCCCTTAC  R: TTCTACAAGCGCAACGGTGT |
| XLOC_055640 | Predicted protein | F: TCCTTGCTCCAGAGTCCAGA  R: CGTGAGCTTGAGCCCCATTA |
| XLOC_002529 | Pathogenesis-related proteins | F: GGACTACCTTTCACCCCACA  R: ATCCTCTGGTTGGCGTAGC |
| XLOC_069634 | CBF protein 4 | F: ACACCACCGCTAAGTCCTC  R: TTAGTGGAGACGCTTGACTGG |
| XLOC_067584 | Pathogenesis-related protein 1 | F: GTAGTCCCCGGCTCGGAAG  R: CCGCCACTTTCATTCGTTGC |
| M36650.1 | GAPDH (actin) | F:AAGCATGAAGATACAGGGAGTGTG  R:AAATTTATTCTCGGAAGAGGTTGTACA |
| AF025292 | *HvHAK1* | F: ATAGGCGATGGAACCTTGAC  R: GACCGAGAAGAGCATGAACA |
