# Supplementary material for: Comparative Transcriptome Profiling of Two Tibetan Wild Barley Genotypes in Responses to Low Potassium
Source: PLoS One. 2014 Jun 20;9(6):e100567. doi: 10.1371/journal.pone.0100567 (PMC4065039; doi:10.1371/journal.pone.0100567)
Supplement: Table S7 — DEGs are involved in Starch and Sucrose metabolism or Cysteine and Methionine metabolism. (DOC) [file pone.0100567.s013.doc]

Table S7. DEGs are involved in Starch and Sucrose metabolism or Cysteine and Methionine metabolism. Line ‘—’ presented in the table means without significant difference in gene expression.

| Gene id | Log2(fold change) | | | | |
| --- | --- | --- | --- | --- | --- |
|  | XZ153 | |  | XZ141 | |
|  | 6h | 48h |  | 6h | 48h |
| **Starch and sucrose metabolism** | | | | | |
| XLOC_059075 | — | 2.11 |  | — | — |
| XLOC_073603 | — | — |  | -2.02 | — |
| XLOC_073751 | — | — |  | -1.90 | — |
| XLOC_032956 | — | 6.31 |  | -5.79 | — |
| XLOC_093329 | — | — |  | — | -2.90 |
| XLOC_064715 | 2.17 | — |  | — | — |
| **Cysteine and methionine metabolism** | | | | | |
| XLOC_053580 | — | — |  | -1.85 | — |
| XLOC_070768 | — | — |  | -2.51 | -2.21 |
| XLOC_053899 | — | — |  | -1.86 | -1.81 |
| XLOC_055421 | — | — |  | -1.89 | -1.72 |
