# Supplementary material for: Comparative Transcriptome Profiling of Two Tibetan Wild Barley Genotypes in Responses to Low Potassium
Source: PLoS One. 2014 Jun 20;9(6):e100567. doi: 10.1371/journal.pone.0100567 (PMC4065039; doi:10.1371/journal.pone.0100567)
Supplement: Figure S6 — DEGs related to low-K tolerance were mapped to the starch and sucrose metabolism pathway. The column marked in color indicates the differentially expressed enzymes. Four small squares from left to right represent different treatment time of the two varieties (XZ153-6h, XZ153-48h, XZ141-6h, XZ141-48h). Gene expression is displayed by different colors and the relative levels of expression are showed by a color gradient from low (blue) to high (red). (PDF) [file pone.0100567.s006.pdf]

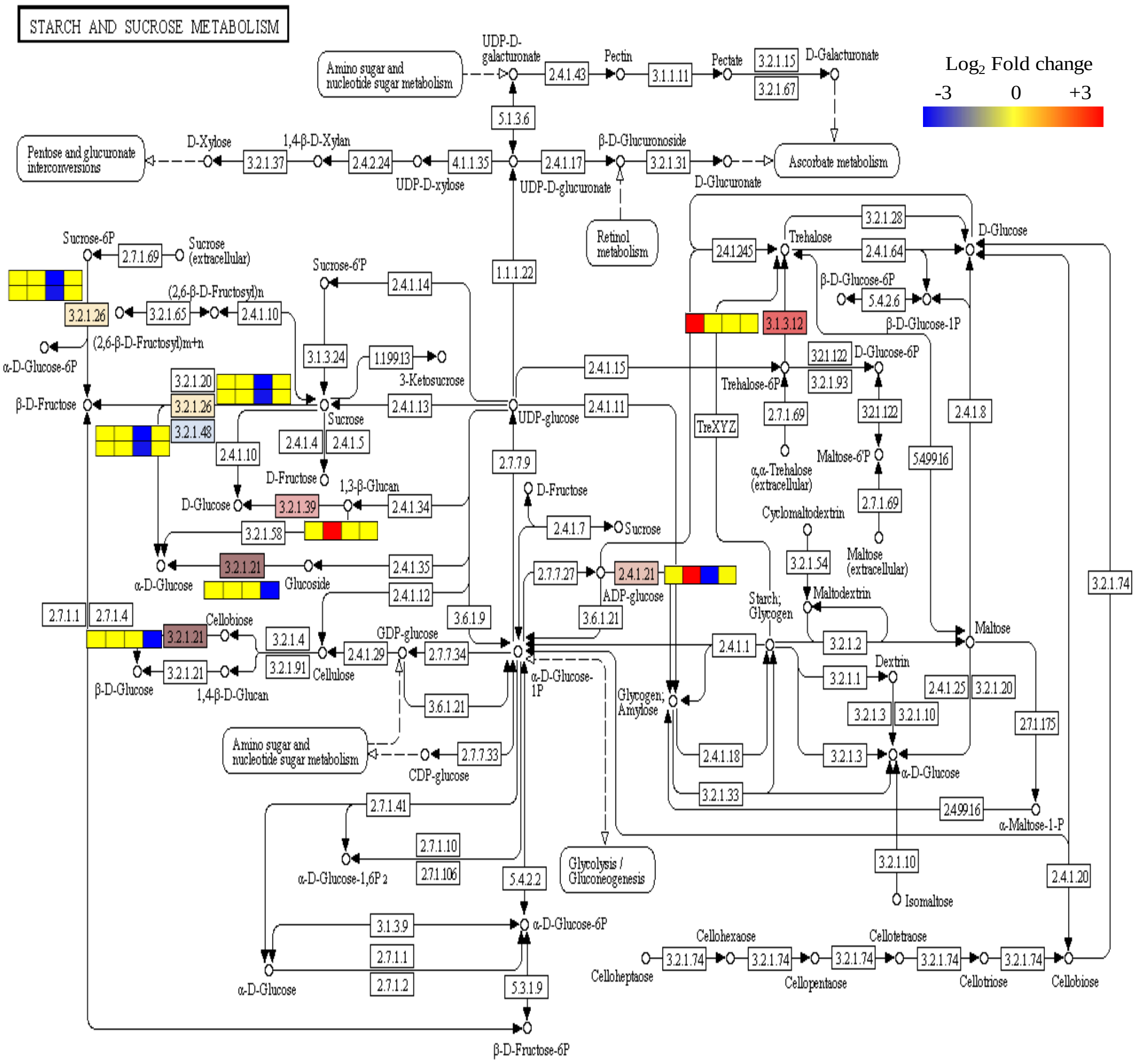

Figure S6. DEGs related to low-K tolerance were mapped to the starch and sucrose metabolism pathway. The column marked in color indicating that enzymes with differentially expressed. Four small squares from left to right represent different treatment time of the two varieties (XZ153-6h, XZ153-48h, XZ141-6h, XZ141-48h). Gene expression is displayed by different colors and the relative levels of expression are showed by a color gradient from low (blue) to high (red).
